# A HALP score-based prediction model for survival of patients with the upper tract urothelial carcinoma undergoing radical nephroureterectomy

**DOI:** 10.17305/bjbms.2021.6543

**Published:** 2021-12-21

**Authors:** Xiaomin Gao, Binwei Lin, Qi Lin, Tingyu Ye, Tao Zhou, Maolin Hu, Honghui Zhu, Feng Lu, Wei Chen, Peng Xia, Fangyi Zhang, Zhixian Yu

**Affiliations:** 1 Department of Urology, The First Affiliated Hospital of Wenzhou Medical University, Wenzhou, Zhejiang, P.R. China; 2 Department of Urology, Rui’an People’s Hospital, The Third Affiliated Hospital of the Wenzhou Medical University, Wenzhou, Zhejiang, P.R. China; 3 Department of Urology, Second Affiliated Hospital, Zhejiang University School of Medicine, Hangzhou, Zhejiang, P.R. China; 4 Department of Transplantation, The First Affiliated Hospital of Wenzhou Medical University, Wenzhou, Zhejiang, P.R. China

**Keywords:** Post-operative survival, HALP, upper tract urothelial carcinoma, prognostic model

## Abstract

The HALP score, which is the combination of hemoglobin, albumin, lymphocyte, and platelets has been confirmed as an important risk biomarker in several cancers. We aimed at evaluating the prognostic value of the HALP score in patients with non-metastatic upper tract urothelial carcinoma (UTUC). In this study, we retrospectively enrolled 533 of the 640 patients from two centers (315 and 325 patients, respectively) who underwent radical nephroureterectomy (RNU) for UTUC. The cutoff value of HALP was determined using the Youden index by performing receiver operating characteristic curve analysis. The relationship between post-operative survival outcomes and pre-operative HALP level was assessed using Kaplan-Meier and Cox regression analyses. As a result, the cutoff value of HALP was 28.67 and patients were then divided into HALP <28.67 group and HALP ≥28.67 group. Kaplan–Meier analysis and log-rank test revealed that HALP was significantly associated with overall survival (OS) (*p* < 0.001) and progression-free survival (PFS) (*p* < 0.001). Multivariate analysis demonstrated that a lower HALP score was an independent risk factor for OS (HR = 1.54, 95% CI, 1.14-2.01, *p* = 0.006) and PFS (HR = 1.44, 95% CI, 1.07-1.93, *p* = 0.020). Nomograms of OS and PFS incorporated with HALP score were more accurate in predicting prognosis than without it. The HALP score could also stratify patients for survival under different pathologic T stages in the subgroup analysis. Therefore, pretreatment HALP score was an independent prognostic factor of OS and PFS in UTUC patients undergoing RNU.

## INTRODUCTION

Upper tract urothelial carcinoma (UTUC) is a rare malignancy, accounting for 5% of urothelial carcinomas [1,2] and affecting up to 2 people/100,000 [3]. More than 50% of UTUCs are muscle-invasive or locally advanced at diagnosis [3], and the urological outcomes of patients with UTUC following radical nephroureterectomy (RNU) are unsatisfactory, including high tumor recurrence rate, high distant metastasis rate, and high mortality [4]. To further facilitate clinical decision-making, it is important to identify the factors that can predict postoperative prognosis in patients with UTUC.

Besides the traditional TNM system, accumulating evidence has demonstrated that hematological parameters, including neutrophil, lymphocyte, monocyte, platelet counts, serum hemoglobin, albumin, and fibrinogen, play an important role in cancer progression and metastasis [5-9]. These inflammatory and nutritional indices have been shown to be closely related to the malignancy degree of cancer and long-term survival in patients with cancer after surgery [10]. The combination of these indexes accurately predicts prognosis than a single index, such as neutrophil-to-lymphocyte ratio, monocyte-to-lymphocyte ratio, and platelet-to-lymphocyte ratio (PLR) [11,12]. Recently, the combination of hemoglobin, albumin, lymphocytes, and platelets (HALP) has been suggested to be a favorable risk predictor of patient survival in several solid tumors, including gastric [13], colorectal [14], pancreatic [15], renal [16], and bladder [17] cancers.

In this study, we aimed to investigate whether pre-operative HALP score could serve as an independent and strong risk factor of overall survival (OS) and progression-free survival (PFS) in UTUC patients.

## MATERIALS AND METHODS

### Description of enrolled patients

**FIGURE 1:**
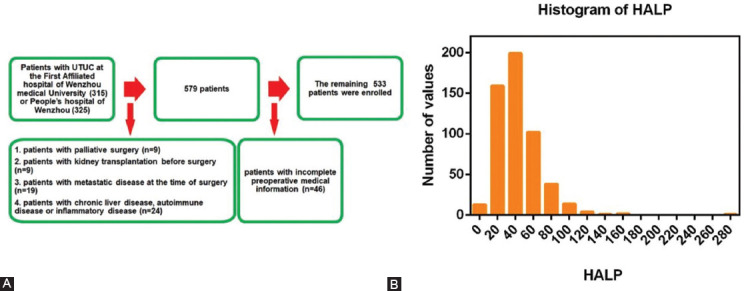
The patient selection flowchart (A) and histogram of HALP (B). HALP: Hemoglobin, albumin, lymphocyte, and platelet.

This study was approved by the ethics committees of The First Affiliated Hospital of Wenzhou Medical University and the Third Clinical Institute Affiliated with Wenzhou Medical University, People’s Hospital of Wenzhou, and informed consent was waived because of its retrospective nature. A total of 640 patients with histologically confirmed non-metastatic UTUC (T1-4N0-1M0) were included in this study. Among them, 315 patients were enrolled from The First Affiliated Hospital of Wenzhou Medical University from March 2005 to August 2015, and 325 patients were recruited from The Third Clinical Institute Affiliated to Wenzhou Medical University, People’s Hospital of Wenzhou from July 2003 to December 2016. The inclusion criteria contained patients who: (1) Underwent curative RNU; and (2) could complete all tests, especially for pre-operative serum platelet and lymphocyte counts, pre-operative hemoglobin, and albumin levels. The exclusion criteria contained patients who: (1) With palliative surgery (n = 9); (2) with kidney transplantation before surgery (n = 9); (3) with metastatic disease at the time of surgery (n = 19); (4) with chronic liver disease, autoimmune disease or inflammatory disease (n = 24); and (5) with incomplete preoperative medical information (n = 46); (Figure 1A). Ultimately, 533 patients were included in this study, and no patient underwent neoadjuvant chemotherapy or radiotherapy preoperatively.

### Data collection

The following variables were collected from the 533 patients: Sex, age, body mass index (BMI), American Society of Anesthesiologists (ASA) grade, hydronephrosis, surgical approach, pre-operative serum platelet and lymphocyte counts, preoperative hemoglobin and albumin levels, chronic kidney disease (CKD) stage, tumor size, tumor site, multifocality, pathologic T stage, N stage, tumor grade, lymphovascular invasion (LVI), and adjuvant therapy after surgery. The American Joint Committee on Cancer TNM Classification (7^th^ edition) and the World Health Organization 1973 grading system were used for tumor staging and tumor grading, respectively. HALP was defined as hemoglobin × albumin × lymphocyte/platelet and PLR as platelet/lymphocyte.

### Follow-up protocol

The post-operative follow-up assessment included blood and urine evaluation, computed tomography or magnetic resonance imaging, and cystoscopy. Patients were examined every 3 months for the 1^st^ year, every 6 months from the third to fifth month, and once per year thereafter. Patient survival information was obtained from medical records, telephone follow-up, or the patients’ social security death index. OS and PFS were used as endpoints of the present study and were measured from the date of surgery until the date of death from any cause, or the date of radiologically or histologically confirmed tumor recurrence, respectively.

### Statistical analysis

Statistical analysis was performed using SPSS software (version 25.0; IBM, Armonk, NY). The optimal cutoff values of HALP and PLR were determined by performing receiver operating characteristic (ROC) curve analysis using the Youden index. The differences in patients’ characteristics were assessed by performing Chi-squared and Student’s *t*-test. The Kaplan-Meier method and log-rank test were applied to compare the survival rate. Univariate and multivariate analyses (forward selection) were performed to identify significant predictors of OS and PFS; variables with *p* < 0.05 in the univariate analysis were selected for multivariate analysis. Nomograms were established based on independent factors (*p* < 0.05) in the multivariate analysis using R software. Calibration plot and concordance index (c-index) were applied to assess the performance of nomograms using R software (version 3.6.0) with rms, Hmisc, and ggplot packages. A bootstrap method with 1,000 resamples was used to validate the nomograms. All *p* values were two-tailed, and *p* < 0.05 was considered statistically significant.

## RESULTS

### Patient characteristics

Of the 533 enrolled patients with non-metastatic UTUC, 369 (69.23%) were men, and 164 (30.77%) were women. The mean age was 66.71 ± 10.4 years, and the median age was 68.00 years (interquartile range 60.00-74.00). A total of 324 (60.79%) patients were older than 65 years, and 209 patients were younger than 65 years. A total of 390 (73.17%) patients had a normal BMI, while 42 (7.88%) patients had CKD at 4-5 stages. Laparoscopic-method RNU was performed in 336 (63.04%) patients, and 197 (36.96%) patients received open-method RNU. There were 314 (58.91%) patients with pelvicalyceal tumors, 191 (35.83%) patients with ureter tumors, and 28 (5.26%) patients with both pelvicalyceal and ureter tumors. The median follow-up time was 39.60 (21.55-64.95) months, with 178 (33.40%) all-cause deaths and 191 (35.83%) patients experiencing tumor recurrence after surgery. The remaining information about patient demographic, pathologic, and survival status features is summarized in Table 1.

**TABLE 1:**
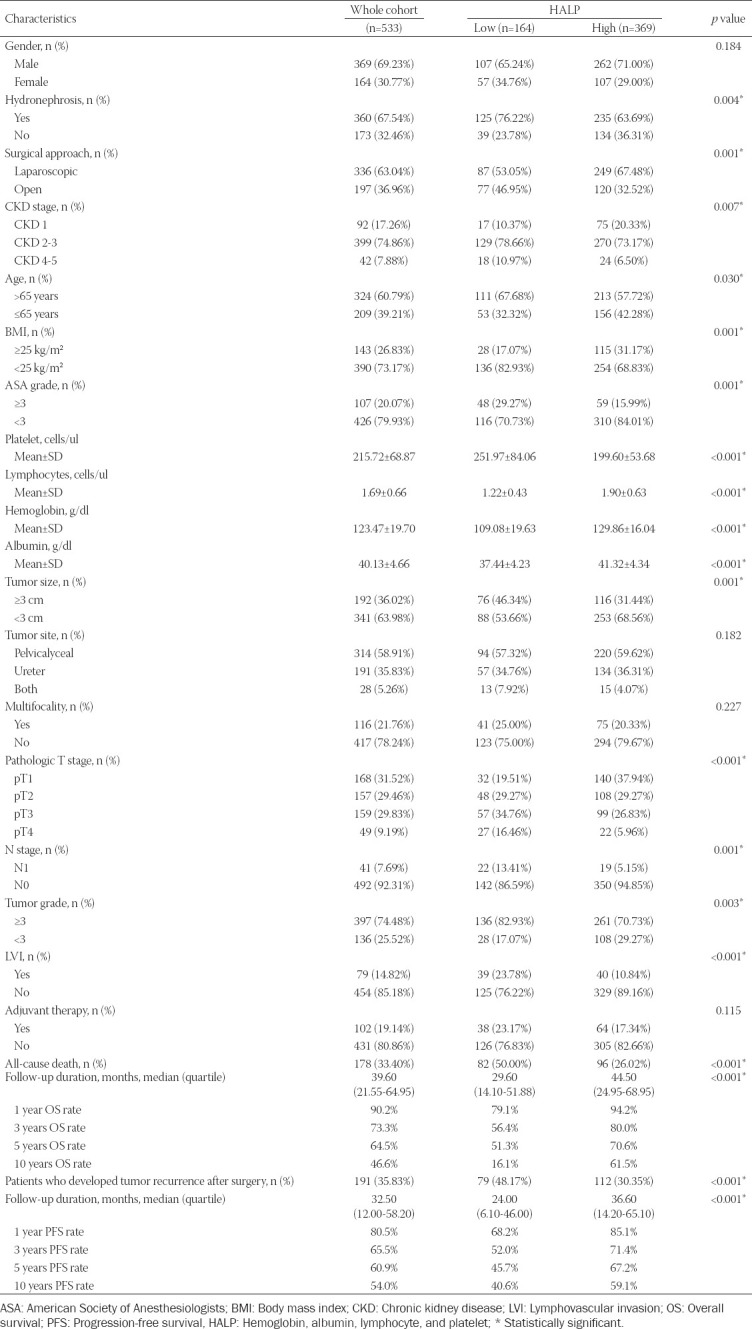
Clinicopathologic characteristics of the whole cohort according to HALP

**SUPPLEMENTARY FIGURE 1:**
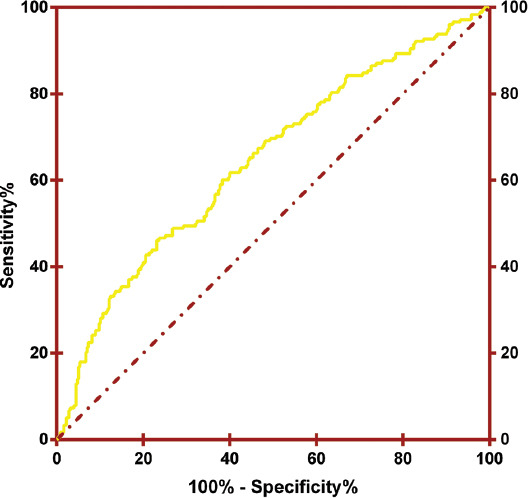
Determination of the optimal cutoff value for HALP based on the ROC analysis. HALP: Hemoglobin, albumin, lymphocyte, and platelet; ROC: Receiver operating characteristic

**SUPPLEMENTARY FIGURE 2:**
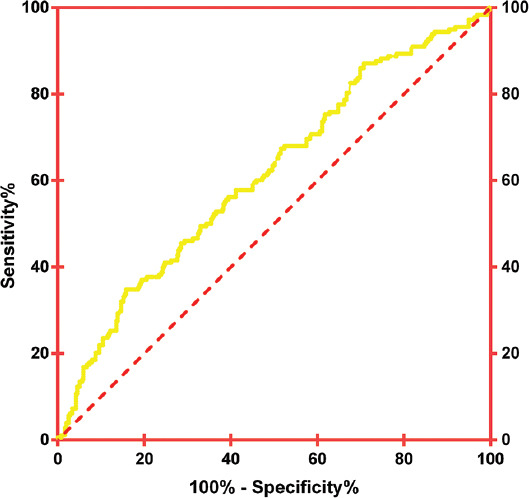
Determination of the optimal cutoff value for PLR based on the ROC analysis. ROC: Receiver operating characteristic; PLR: Platelet-to-lymphocyte ratio

The minimum, median (quartiles), and maximum HALP levels were 4.91, 38.79 (25.99-56.51), and 270.47, respectively. In addition, Figure 1B shows the histogram of HALP. The ROC curve analysis showed that the optimal cutoff value of HALP was 28.67 (Supplementary Figure 1). The area under the curve (AUC), sensitivity, specificity, and Youden index were 0.64 (0.59-0.69), 76.90%, 53.90%, and 0.308, respectively. The optimal cutoff value, AUC, sensitivity, specificity, and Youden index of PLR were 179.89, 0.61 (0.56-0.67), 59.55%, 54.65%, and 0.142, respectively (Supplementary Figure 2). Subsequently, the entire population was divided into patients with low HALP levels (n = 164, 30.77%) and patients with high HALP levels (n = 369, 69.23%). Table 1 shows that aging, lower BMI, higher ASA grade, the presence of hydronephrosis, laparoscopic surgery, and higher CKD stage were frequently observed in the low HALP group than in the high HALP group. Furthermore, patients with low HALP levels were more likely to have higher platelet counts, lower lymphocyte counts, lower serum hemoglobin and albumin levels, large tumor size, higher pathologic T stage, tumor grade, and the presence of LVI and positive nodes (all *p* < 0.05). There was no significant difference between the two groups with respect to sex, multifocality, or adjuvant therapy (all *p* > 0.05).

### Association of HALP score with patient outcomes

**FIGURE 2:**
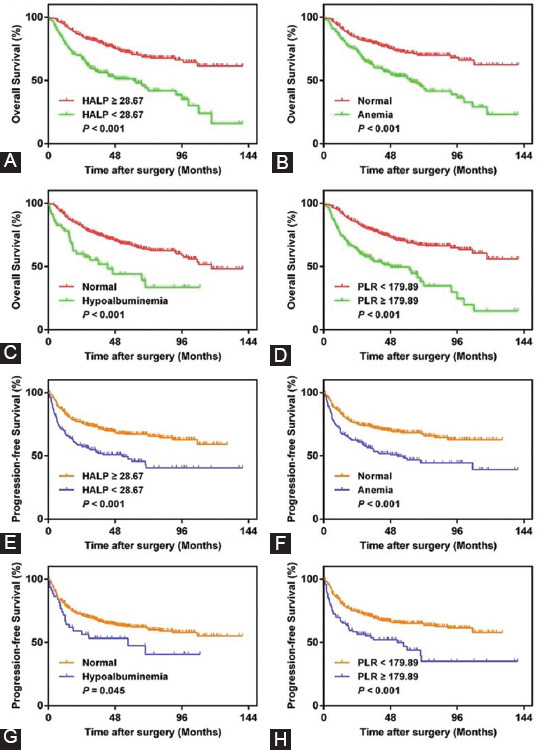
Kaplan–Meier analysis for OS (A-D) and PFS (E-H) in UTUC patients according to HALP, hemoglobin, albumin, and PLR. OS: Overall survival; PFS: Progression-free survival; HALP: Hemoglobin, albumin, lymphocyte, and platelet.

Kaplan–Meier curves and log-rank test revealed that low HALP score, albumin, hemoglobin, and high PLR were significantly associated with worse OS and PFS (all *p* < 0.05) (Figure 2). The low HALP group had shorter 1-, 3-, 5-, 10-year OS rate, and PFS rate compared with high HALP group (OS: 79.1%, 56.4%, 51.3%, 16.1% vs. 94.2%, 80.0%, 70.6%, 61.5%, respectively; PFS: 68.2%, 52.0%, 45.7%, 40.6% vs. 85.1%, 71.4%, 67.2%, 59.1%, respectively).

Univariate analysis demonstrated that aging, lower BMI, presence of hydronephrosis, open surgical approach, lower HALP (OS: HR = 2.45, 95% CI, 1.82-3.30, *p* < 0.001; PFS: HR = 1.98, 95% CI, 1.48-2.64, *p* < 0.001) PLR, the presence of anemia and hypoproteinemia, higher CKD stage, larger tumor size, tumor presence both in pelvicalyceal and ureter, presence of multifocality, higher pathologic T and N stage, higher tumor grade, presence of LVI, and history of receiving adjuvant therapy were significantly associated with poorer OS or PFS (all *p* < 0.05) (Tables 2 and 3). Subsequently, multivariate analysis showed that age and tumor size were significant factors of OS. Pathologic T stage, N stage, tumor grade, and adjuvant therapy were significantly correlated with OS and PFS. As expected, HALP score was identified as an independent risk factor for OS (HR = 1.54, 95% CI, 1.14-2.01, *p* = 0.006) and PFS (HR = 1.44, 95% CI, 1.07-1.93, *p* = 0.020).

**TABLE 2:**
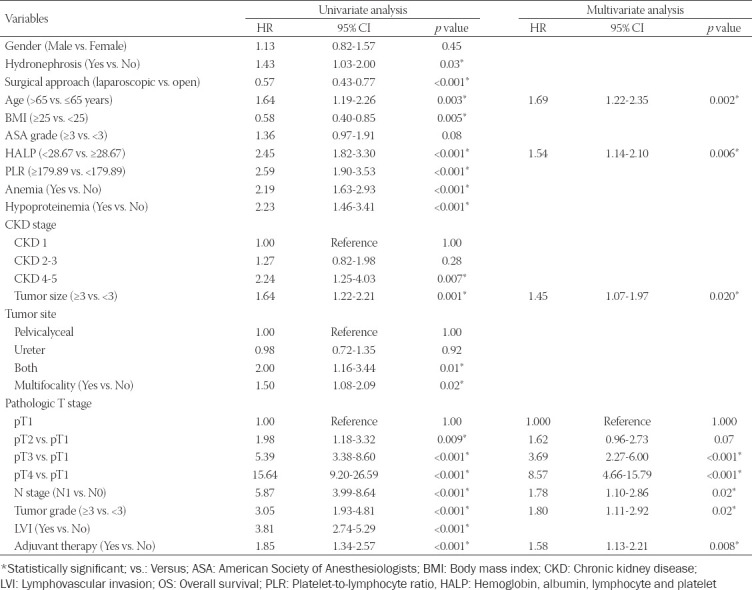
Univariate and multivariate analysis of variables for the prediction of OS

**TABLE 3:**
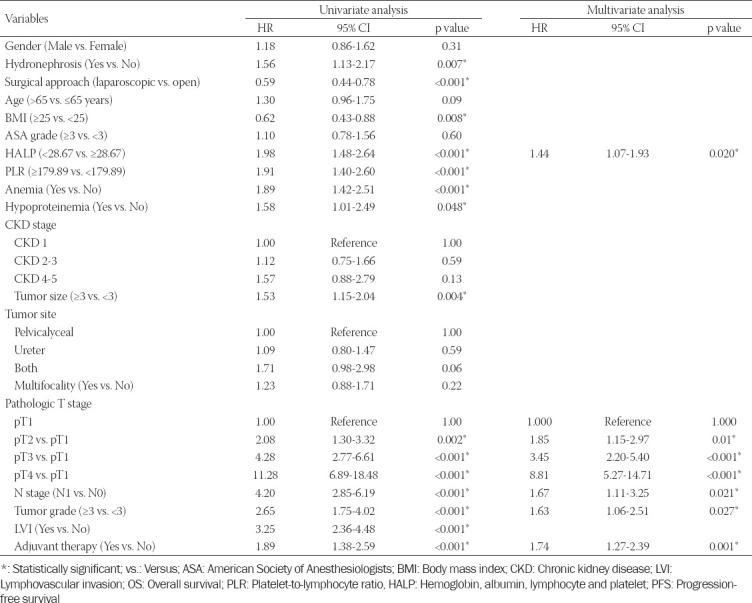
Univariate and multivariate analysis of variables for the prediction of PFS

### HALP score based risk model for OS and tumor progression after curative surgery

**FIGURE 3:**
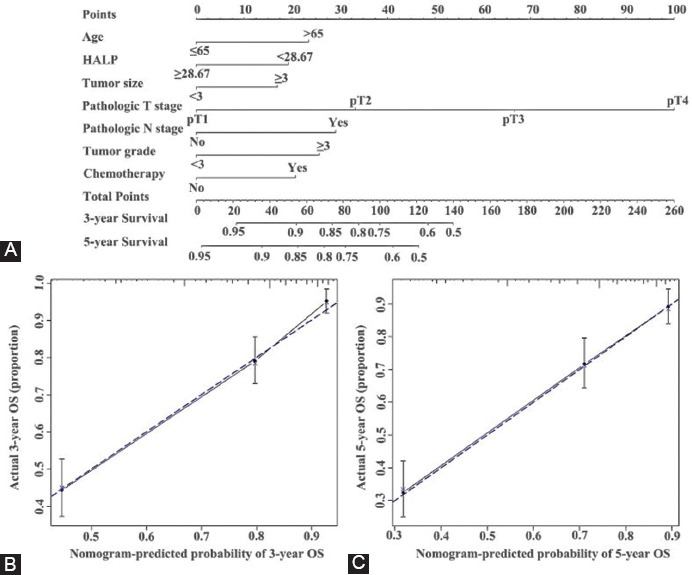
Established nomograms (A) for OS in patients with UTUC and calibration curve for predicting 3- and 5-year; (B, C) survival of OS. To use the nomogram, an individual UTUC patients’ value is located on each variable axis, and a line is depicted upward to determine the number of points received for each variable value. Subsequently, the sum of these numbers is located on Total Point axis, and a line is drawn downward to the survival axes to determine the likelihood of 3- and 5-year survival. OS: Overall survival; UTUC: Upper tract urothelial carcinoma.

**FIGURE 4:**
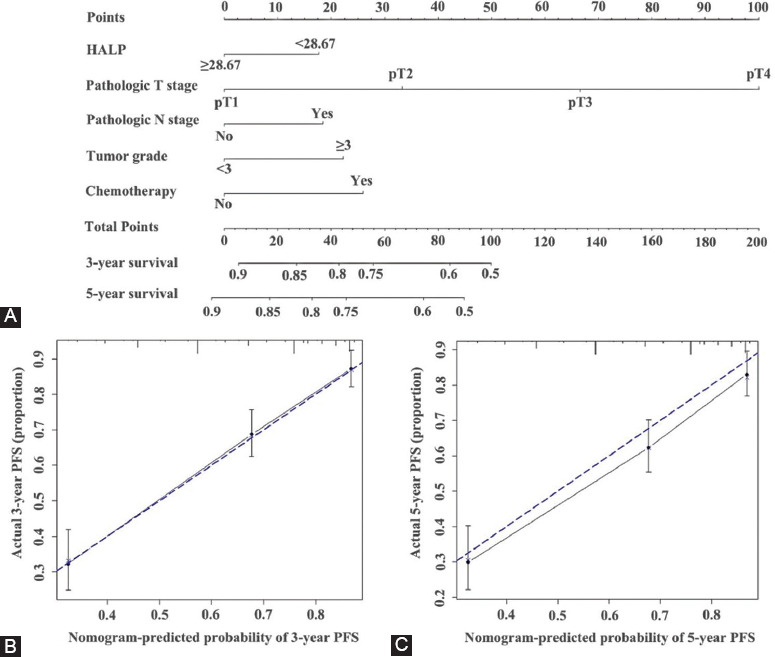
Established nomograms (A) for PFS in patients with UTUC and calibration curve for predicting 3- and 5-year; (B, C) survival of PFS. UTUC: Upper tract urothelial carcinoma; PFS: Progression-free survival.

**FIGURE 5:**
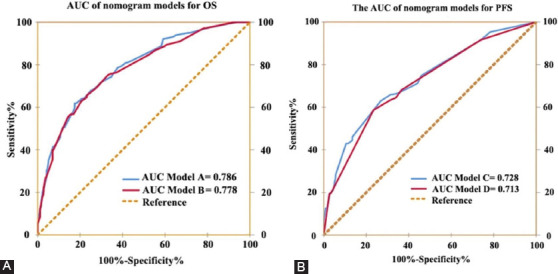
ROC analysis of the prognostic accuracy of HALP for OS and PFS in established models. ROC: Receiver operating characteristic; HALP: Hemoglobin, albumin, lymphocyte and platelet; OS: Overall survival; PFS: Progression-free survival.

We developed nomograms to predict 3 and 5 year OS and PFS for individuals based on HALP score and other variables identified as significant risk predictors in the multivariate analysis (Figures 3A and 4A). The calibration curves of the nomograms for OS and PFS showed that the predicted 3- and 5-year survival was similar to the actual 3- and 5-year survival (Figure 3B and C, Figure 4B and C). The c-indexes (Table 4) and AUC (Table 5 and Figure 5) of nomograms for OS and PFS increased when incorporating HALP into developed models. Hence, the established nomograms, including pathologic T stage, N stage, and HALP, had favorable predictive accuracy compared with traditional predictive tools.

**TABLE 4:**
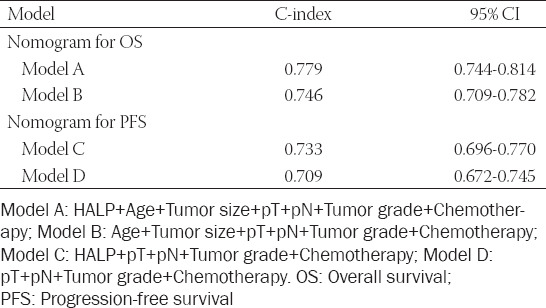
Predictive ability comparison of models for OS and PFS with 1000 bootstraps

**TABLE 5:**
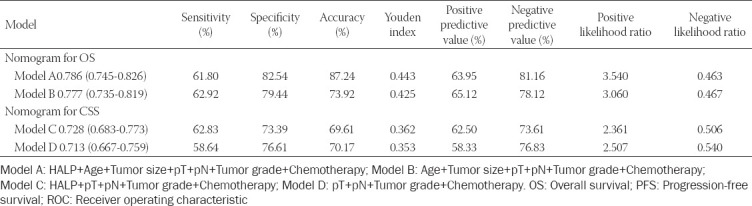
Predictive ability comparison of models for OS and PFS with ROC analysis

### Predictive value of HALP under adjusted pathologic T stage

**FIGURE 6:**
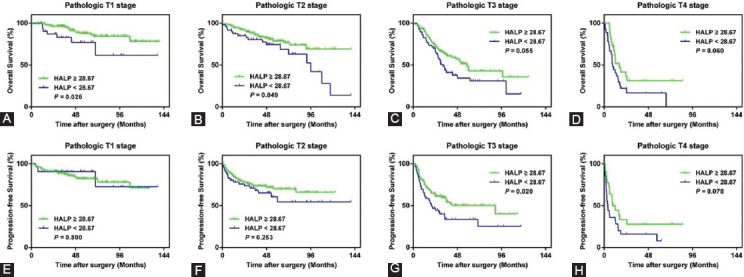
Subgroup analysis to evaluate the prognostic ability of HALP in predicting OS (A-D) and PFS (E-H) in UTUC patients under pathologic T stage. HALP: Hemoglobin, albumin, lymphocyte, and platelet; OS: Overall survival; PFS: Progression-free survival.

Among patients with pT1-2 stage tumors, those with low HALP levels had significantly worse OS than those with high HALP levels (*p* = 0.03 for pT1, *p* = 0.049 for pT2) (Figure 6). However, PFS did not significantly differ between the two groups (*p* = 0.80 for pT1, *p* = 0.25 for pT2). Among patients with pT3-4 stage tumors, patients in the low HALP group had significantly worse PFS (*p* = 0.02 for pT3) compared with patients in the high HALP group and had a trend of poorer OS (*p* = 0.06 for pT3, *p* = 0.06 for pT4) and PFS (*p* = 0.08 for pT4), although the differences were not significant.

## DISCUSSION

Many previous studies have demonstrated that malnutrition status and systemic inflammatory response are associated with each process of cancer initiation, progression, and metastasis. The HALP score, which consists of hemoglobin, albumin, lymphocytes, and platelets, is a newly established scoring tool for representing the status of both host inflammation and nutrition. In this study, our results revealed that the HALP score was statistically correlated with aging, larger tumor size, pathologic T and N stage, tumor grade, LVI, and other clinical parameters indicative of an aggressive phenotype. Multivariate analysis identified HALP score as a significant predictor of OS and PFS in patients with UTUC following RNU.

Anemia is a common symptom in patients with cancer, which results from chronic blood loss, iron, Vitamin B12, or folate deficiency [16], and imbalanced inflammation regulation [18]. Cancer-related anemia is associated with poor performance status and quality of life, increased clinical symptoms, and decreased tolerance, and recovery ability of surgery and chemotherapy [19]. The previous studies have noted that hemoglobin deficiency could contribute to low response to treatment, tumor progression, and unfavorable survival outcomes in cancer patients [20,21]. Serum albumin was synthesized in the liver and could be affected by systemic factors, including inflammation and stress. As an important indicator of a patient’s inflammatory and nutritional status, low albumin levels are believed to predict poor outcomes in various cancers, including UTUC [22]. A tumor-related systemic inflammatory response is one of the hallmarks of cancer [23]. The infiltration of inflammatory cells, including lymphocytes and platelets in the microenvironment of tumor cells, will exert conflicting effects on tumor initiation and progression. Lymphocytes can inhibit tumor cell proliferation, invasion, and metastasis by initiating and enhancing immune surveillance [24]. The HALP score, which is the integration of these four hematological indexes, is a powerful risk predictor with higher accuracy in predicting OS and PFS for UTUC patients than hemoglobin, albumin, or PLR alone. Therefore, the prediction model was developed and was further determined as an independent factor for the prognosis of patients with UTUC after surgery.

Our study has supported the following points: First, the predictive ability of HALP was confirmed in an independent cohort. Our data were representative and reliable because patients were from two hospitals, which were the largest two urologic centers with the largest sample size for UTUC patients in the south of Zhejiang Province. The predictive ability of the HALP score for UTUC was not better than for renal [16] or bladder [17] cancers. Second, this new biomarker is advantageous because it can be measured preoperatively based on routine laboratory examination, as it is non-invasive, affordable, highly reproducible, and easy to assess compared with tissue-based prognostic biomarkers. Third, the HALP score will help urologists better stratify patients and guide the therapeutic strategies to improve the prognosis. In this study, Table 1 shows that patients with the lower HALP score are more likely to have lower BMI, lower serum hemoglobin, and albumin, which indicate malnutrition. Therefore, adequate amino acid supplementation and physical activities will be recommended for them before radical RNU to improve their HALP score. Fourth, we performed subgroup analysis to gain a better understanding of the prognostic impact of HALP score under pathologic T stages. Lower HALP score patients under pT1-2 stage had significantly poorer OS, as well as for PFS in patients under pT3 stage. However, the low HALP score group had a worse OS (*p* = 0.055) trend under the pT3 stage, and worse OS (*p* = 0.060) and PFS (*p* = 0.078) trends at the pT4 stage, even though these differences were not significant due to the small sample size. Therefore, more patients with a high pT stage should be included in subsequent studies to further assess the prognostic impact of the HALP score on survival outcomes. Furthermore, there was no significant difference for patients with low HALP scores and high HALP scores under pT1 (*p* = 0.80) and pT2 (*p* = 0.25) with regard to PFS. Patients with pT1-2 generally have a long survival time after RNU. Therefore, we suggest that the prognostic value of HALP for PFS under the pT1-2 stage should be further evaluated by performing an investigation of a longer follow-up period.

The major limitations of this study are as follows: First, this retrospective design will increase the bias of population choice. Second, there is no consensus on the cutoff value of the HALP score because the researches focusing on HALP is limited. Third, we did not include patients with metastasis before surgery, and the findings cannot be generalized to all UTUC patients. Furthermore, the effects of dynamic changes in HALP on long-term survival remain to be evaluated to have a better understanding of the association. Therefore, a prospective study with large sample size is needed to validate the results.

## CONCLUSION

Our data suggested that pre-operative HALP score was an independent risk factor for OS and PFS in patients with non-metastatic UTUC after RNU. The developed nomograms based on the HALP score could be used for risk stratification of individual UTUC patients and for choosing a treatment strategy.
